# Sesame Oil-Based Nanostructured Lipid Carriers of Nicergoline, Intranasal Delivery System for Brain Targeting of Synergistic Cerebrovascular Protection

**DOI:** 10.3390/pharmaceutics13040581

**Published:** 2021-04-19

**Authors:** Mohammed A. S. Abourehab, Ahmed Khames, Samar Genedy, Shahin Mostafa, Mohammad A. Khaleel, Mahmoud M. Omar, Amani M. El Sisi

**Affiliations:** 1 Department of Pharmaceutics, Faculty of Pharmacy, Umm Al-Qura University, Makkah 21955, Saudi Arabia; maabourehab@uqu.edu.sa; 2 Department of Pharmaceutics and Industrial Pharmacy, Faculty of Pharmacy, Minia University, Minia 61511, Egypt; 3 Department of Pharmaceutics and Industrial Pharmacy, College of Pharmacy, Taif University, P.O. Box 11099, Taif 21944, Saudi Arabia; 4 General Authority for Health Insurance in Egypt, Giza Branch, Ministry of Health, Giza 12556, Egypt; s.gnedy3@gmail.com; 5 Department of Pharmaceutics, Faculty of Pharmacy, AIN SHAMS University, Cairo 11566, Egypt; mhshain@uqu.edu.sa; 6 Department of Clinical Pharmacy, School of Pharmaceutical Sciences, Universiti Sains Malaysia, Minden, Penang 11800, Malaysia; M.khaleel@student.usm.my; 7 Department of Pharmaceutics and Industrial Pharmacy, Deraya University, Minia 61768, Egypt; mahmoud.omar@deraya.edu.eg; 8 Department of Pharmaceutics and Clinical Pharmacy, Faculty of Pharmacy Sohag University, Sohag 82524, Egypt; 9 Department of Pharmaceutics and Industrial Pharmacy, Faculty of Pharmacy, Beni-Suef University, Beni-Suef 62514, Egypt; amany.elsese@pharm.bsu.edu.eg

**Keywords:** nicergoline, nanostructured lipid carriers, dementia, Box–Behnken design, sesame oil, cognitive function disorders, intranasal drug delivery, nose-to-brain drug targeting

## Abstract

Nicergoline (NIC) is a semisynthetic ergot alkaloid derivative applied for treatment of dementia and other cerebrovascular disorders. The efficacy of sesame oil to slow and reverse the symptoms of neurodegenerative cognitive disorders has been proven. This work aimed to formulate and optimize sesame oil-based NIC-nanostructured lipid carriers (NIC–NLCs) for intranasal (IN) delivery with expected synergistic and augmented neuroprotective properties. The NIC–NLC were prepared using sesame oil as a liquid lipid. A three-level, three-factor Box–Behnken design was applied to statistically optimize the effect of sesame oil (%) of the total lipid, surfactant concentration, and sonication time on particle size, zeta potential, and entrapment efficacy as responses. Solid-state characterization, release profile, and ex vivo nasal permeation in comparison to NIC solution (NIC–SOL) was studied. In vivo bioavailability from optimized NIC–NLC and NIC–SOL following IN and IV administration was evaluated and compared. The optimized NIC–NLC formula showed an average particle size of 111.18 nm, zeta potential of −15.4 mV, 95.11% entrapment efficacy (%), and 4.6% loading capacity. The NIC–NLC formula showed a biphasic, extended-release profile (72% after 48 h). Permeation of the NIC–NLC formula showed a 2.3 enhancement ratio. Bioavailability studies showed a 1.67 and 4.57 fold increase in plasma and brain following IN administration. The results also indicated efficient direct nose-to-brain targeting properties with the brain-targeting efficiency (BTE%) and direct transport percentage (DTP%) of 187.3% and 56.6%, respectively, after IN administration. Thus, sesame oil-based NIC–NLC can be considered as a promising IN delivery system for direct and efficient brain targeting with improved bioavailability and expected augmented neuroprotective action for the treatment of dementia.

## 1. Introduction

Due to the advances in health care and social services provided by modern and industrial countries for their citizens, the average life span has increased with significant rises in elderly populations. This results in an expected increase in old-age diseases, mainly dementia [1,2]. The prevalence of dementia among the elderly population has not only increased in modern and advanced countries but in developing countries as well [3]. It is anticipated that this remarkable spread of dementia will reach 63 million people worldwide by 2030 [4].

For cognitive function disorders (CFDs), the *Diagnostic Statistical Manual of Mental Disorders* (DSM-5) [5] recognizes dementia as a major neurocognitive disorder (NCD) characterized by impairment in a at least a single cognitive domain including attention, memory, and/or language in a progressive manner, resulting in deterioration of regular, normal life activities. Neurocognitive disorder may be mild or major NCD (dementia) [6,7]. Alzheimer’s disease (AD), ischemic (vascular) dementia (VD), frontotemporal dementia, and dementia due to the fact of Parkinson’s disease are the most common forms of dementia [8,9].

Based on etiology and pathological progression, dementia is classified as cortical that occurs due to the atrophy of the neuronal cortex and subcortical that involve vasculature, thalamus, and/or basal ganglia disease [10]. Cortical dementias are mainly progressive, degenerative, and associated with clear cognitive impairment signs of aphasia, apraxia, agnosia, in addition to memory deterioration [11,12]. 

Management of dementia is a challenging and complicated process, but, generally, it is supportive treatment to reduce the suffering of cognitive/behavioral symptoms and delay further progression. Pharmacologic and non-pharmacologic treatment schemes are approved and applied [13,14,15]. Cholinesterase inhibitors are approved by the Food and Drug Administration (FDA) for treatment of cognitive symptoms of dementia [16,17]. These drugs lack the ability to reverse the degenerative effects of the disease [18].

Nicergoline (NIC) is a semisynthetic derivative of ergot alkaloid. Chemically, it is 8-β-(5-bromonicotinoylhydroxymethyl)-1,6-dimethyl-10α-metoxyergoline. It is registered in more than fifty countries for the treatment of age-related cerebrovascular disorders [9,19]. Nicergoline has a selective, potent agonistic effect on α1-adrenergic receptors resulting in vasodilatation and improvement in cerebral circulation, especially in the middle and anterior cerebral arteries in addition to the right occipital lobe [20,21]. Nicergoline also works through versatile patho-physiological mechanisms and has a broad-spectrum action including increasing acetylcholine, noradrenaline, and dopamine release and enzymatic turnover at the nerve endings, resulting in enhanced cholinergic and catecholaminergic neurotransmitter signals, supporting phosphoinositide–PKC translocation and nerve growth factor to combat amyloid deposition, platelet phospholipase antagonistic effect, increasing cerebral oxygen and glucose levels via supporting different cerebral metabolic pathways. It also has antioxidant and neuroprotective characteristics; this allowed its use in the treatment of dementia not only to prevent further impairment of cognitive functions, but it also caused remarkable improvement [22,23,24,25,26]. It was established that approximately 89% of patients under NIC treatment showed significant improvement in behavioral and cognitive functions within two months and continual enhancement up to one year. Vigilance and processing of information also improved within one to two months. In addition, 44–78% improvement in concomitant balance disorders was observed [4]. Nicergoline is also acceptable as a supportive treatment in glaucoma, benign prostatic hyperplasia, dysphagia, pneumonia, and cerebrovascular and peripheral vascular disorders [4,22,27,28].

After oral administration, NIC is rapidly and almost completely absorbed and reaches maximum plasma concentration within three hours. It is highly protein bound (>90%), with an elimination half-life of 15 h, high volume of distribution (exceeded 105 L), and low oral bioavailability (<5%) due to the extensive lover enzymatic degradation [29]. Three metabolic pathways are reported, namely, hydrolysis of ester linkage to produce 1-methyl-10 alpha-methoxy-9,10-dihydrolysergol (MMDL), active metabolite, and demethylation and glucuronide conjugations represent minor metabolic pathways [22,30]. Nicergoline is a BCS class-II drug with poor water solubility (0.002 mg/mL) [31,32].

Recently, it has become well known and approved that AD and VD are rarely to occur separately in pure form but usually overlap and defined as mixed dementia (MD) [33]. An elevated cerebrospinal-fluid (CSF)-to-albumin ratio was reported as a main cause of hardening of cerebral arterioles in VD. With aging, significant alterations occur in the blood–brain barrier (BBB) permeability that play a remarkable pathological role in vascular cognitive impairment [34,35]. Several imaging and pathological studies correlated the BBB disruption and increased permeability with cerebral vascular diseases and elevated amyloid proteins level in basal lamina and tight junctions of the BBB result in impairment of cognitive functions and dementia [36,37,38].

Sesame oil is an edible vegetable fat derived by pressing sesame plant seeds. It has antioxidant, antimicrobial, and anti-inflammatory action. Sesamol, sesamin, and sesamolin are the major and most common antioxidant ligands in the oil. Anti-apoptotic, anti-hyperlipidemic, and anti-hypertension properties are also reported [39,40]. In brain, they reduce peroxidation of lipids, support oxidative enzymes, and decrease the neuroinflammatory markers. Hence, preventing disruption of cerebrovascular endothelial cells and maintaining the BBB’s integrity, in addition to reversing the neuronal damage effects of ischemia and/or other oxidative stress injuries [41,42,43,44]. VanGilder and Huber [45] reported that sesamol significantly retarded the BBB dysfunction progression and reversed the symptoms of mild cognitive disorders in diabetic patients. In another in vitro study on cultured astrocytes, sesamol significantly lowered cellular hydrogen peroxide and nitric oxide production to maintain the BBB’s integrity. It also diminished the hyperactivity of the monoamine oxidase enzyme associated with the neurodegenerative disorders of Alzheimer’s disease, stroke, and other age-related cerebral disorders [46].

The BBB of cerebral blood vessels is a complicated, dynamic structure formed due to the interactions among different cell types collectively described in terms of a neurovascular unit, which is very important for maintaining normal cerebral functions and controlling the transfer of fluids and different molecules from systemic circulation to the brain. This makes efficient treatment of CNS disorders a difficult and complicated process [29,47].

To improve drug permeability through the BBB, some invasive techniques to make transient opening of the BBB were studied; high cost and increased risk of infection in comparison to efficacy was reported [48,49,50]. Thus, presence of a non-invasive brain targeting techniques became a significant and challenging research interest to ensure efficient and safe drug therapies for CNS disorders. Recently, intranasal (IN) drug delivery has emerged as an efficient, alternative non-invasive route of administration able to bypass the BBB, transferring drugs directly to the brain and CNS through the olfactory and trigeminal neuronal pathways [51,52]. It has the advantages of being safe, non-invasive, self-administered with rapid onset of action, escaping first-pass enzymatic degradation in the liver with improved bioavailability. Small formulation volume, limited absorption of hydrophilic and high molecular weight drugs in addition to short residence time due to the rapid washing out by nasal mucociliary represent the main limitations for this administration route [53,54,55,56].

Nanotechnology is a promising strategy for brain targeting, as it allows application of several techniques and uses a variety of biocompatible nanocarriers for direct and efficient brain targeting. In addition, it can be easily functionalized with a specified brain-targeting ligand to guarantee optimum CNS selectivity. Polymeric nanocarriers, nanoemulsions, liposomes, solid lipid nanoparticles, and nanostructured lipid carriers (NLCs) have been recently studied and considered as reliable, promising nose-to-brain delivery systems [57,58].

When compared to polymeric nanoparticles, lipid nanoparticles show rapid cellular uptake into cerebral tissue, low cytotoxicity due to the biocompatible and biodegradable composition, and they can also protect the drug from enzymatic nasal degradation and carry the possibility of large-scale production. In addition, cytotoxic data and safety profiles for these systems are well defined. Nanostructured lipid carriers can be considered as the enhanced 2nd generation lipid nanocarrier systems of solid lipid nanoparticles. For that, liquid lipid was incorporated within the lipid matrix in different ratios. This added the advantage of higher drug loading capacity, extended shelf-life stability, and lessened the bursting effect, which allows controlling the drug release [59,60].

In this work, NIC was formulated into NLC systems for intranasal drug delivery using sesame oil with expected synergistic and augmented neuroprotective properties. Nicergoline formulations were optimized for higher entrapment capacity, small particle size, and optimum zeta potential. The optimized NIC–NLC formula was fully evaluated. Ex vivo permeation and in vivo bioavailability studies in comparison to drug solution was also investigated in animal models.

## 2. Materials and Methods

### 2.1. Materials

Nicergoline and tizanidine hydrochloride (purchased from Sigma–Aldrich, Steinheim, Germany), Precirol ATO 5 (gift from Gattefosse SAS, Saint-Priest Cedex, France). Tween 80, and dicetyl phosphate (Merck KGaA, Darmstadt, Germany). All other solvents were HPLC grade (Sigma–Aldrich, Steinheim, Germany).

### 2.2. Methodology

#### 2.2.1. Experimental Design

To statistically optimize the selected NLC formulation variables and based on their number and proposed levels, the Box–Behnken statistical design with three levels and three factors was applied using Design-Expert 8.0.3 software (Stat-Ease Inc., Minneapolis, MN, USA). A matrix of seventeen runs (NIC–NLC formulae) was constructed to optimize the additive linear and nonadditive interactive effects of independent nominated formulation variables, namely, sesame oil (%) of the total lipid (A), surfactant concentration (B), and sonication time (ST, C) on particle size (PS, R_1_), zeta potential (ZP, R_2_), and entrapment efficacy (EE%, R_3_) as dependent variables (responses). The independent variables with their levels and the responses are presented in Table 1. Polynomial equations of the best fitted model were generated depending on higher determination coefficients and significance value at selected probability levels, ANOVA test was applied to test the validity of the collected data. Results were illustrated by 3D and perturbation plots. 

#### 2.2.2. Validation of the Experimental Method

To investigate the validity of the optimization procedure, the numerical prediction optimization function afforded by software was applied within the proposed constraint levels of selected responses. Three optimized NIC–NLC formulae (checkpoints) with higher desirability were prepared and evaluated for different response values. The observed and predicted values were compared to calculate the prediction errors (%).

### 2.3. Preparation of NIC–NLCs

The NIC–NLC formulae were prepared using a hot-emulsification-ultrasonication technique [61]. A mixture of Precirol (as solid lipid) and sesame oil (as liquid lipid) was melted at 70–80 °C to obtain a clear homogenous oily phase. The drug (0.5%) and 0.03% *w/v* dicetyl phosphate (surface charge modifier) [62] were dispersed in hot melt with continuous stirring. Aqueous Tween 80 solution (1%, 2%, and 3%) was prepared and heated to the same temperature. The oily melted phase was slowly dispersed into the hot aqueous surfactant solution during stirring to obtain coarse emulsion. The latter was further homogenized at (5000 rpm at 80 °C) for 15 min. The resulting pre-emulsion was then subjected to 400 W power ultrasonication for different time intervals (namely, 2, 4, and 6 min) using a probe-sonicator (Baldelin, Berlin, Germany) in a water bath. The resultant nanoemulsion was dipped in an ice bath to affect the crystallization of lipids and formation of NIC–NLC. 

### 2.4. Characterization of the Prepared NIC–NLCs

The prepared NIC–NLC formulae were evaluated and optimized by measuring the following.

#### 2.4.1. Particle Size (PS) and Size Distribution

After suitable dilution with double distilled water, the average diameter, and Polydispersity Index (PDI) of the prepared NIC–NLC were investigated at a fixed light scattering angle of 90 °C using Zetasizer-4, Malvern instruments (Malvern, Worcestershire, UK) at 25 °C. Each measurement was repeated three times, and the average of the three readings was recorded. 

#### 2.4.2. Entrapment Efficiency (EE%) and Loading Capacity (LC)

A sample (2.5 mL) of undiluted NIC–NLC was packed in the outer tube of the Centrisart apparatus and centrifugated at 10,000 rpm for 30 min. The aqueous solution in the recovery chamber was collected and assayed for free NIC content using a suitable HPLC assay method [63]. The entrapment efficiency (EE) was calculated as:

(1) $$\mathrm{EE}\left(\%\right)=\left(\frac{\mathrm{DI}-\mathrm{DF}}{\mathrm{DI}}\right)\;\times \;100$$

(2) $$\mathrm{LC}=\left(\frac{\mathrm{DI}-\mathrm{DF}}{\mathrm{WP}}\right)\;\times \;100$$

DI = Initial NIC concentration. DF = Concentration of free NIC in the aqueous phase. WP = Weight of nanoparticles.

#### 2.4.3. Zeta Potential (ZP) 

The surface charge of the prepared NLC particles was measured in terms of zeta potential (ZP) using the zeta-nanoparticle electrophoresis analyzer, Malvern Zetasizer-4 (Nano ZS, Zen 3600, Malvern Instruments Limited, UK) at 25 °C after suitable dilution with double distilled water in suitable intensity at pH 6.5 ± 0.5. The average of three determinations was always considered.

#### 2.4.4. Determination of the NIC Release Rate from the Optimized NLC Formulations

The NIC release pattern from optimized NLC formulations was performed at simulated nasal conditions according to pharmacopeial standards using the dialysis bag diffusion technique [64] in simulated nasal fluid (SNF) composed of sodium chloride 0.745 g, potassium chloride 0.129 g, and calcium anhydrous chloride 0.005 g and purified water to 100 mL, pH 6.4 [65]. 

A 1 mL sample of each optimized NIC–NLC formula (equivalent to 10 mg NIC) and NIC–Sol (10 mg) was separately placed into a dialysis bag (12 kDa-MWCO, 2.4 nm pore size) hydrated by soaking in SNF for 24 h before use. These bags were directly immersed in (200 mL) SNF, to maintain sink conditions, maintained at 37 ± 0.5 °C and stirred at 50 rpm. At predetermined time intervals, 3 mL sample aliquots were withdrawn (with replacement with fresh SNF) for 6 h and analyzed for their drug content using an HPLC assay method [63]. The mean of six determinations was considered.

##### Kinetic Modeling of Release Data

For quantitative determination of release mechanism of NIC from optimized NLC formula, release data were mathematically analyzed for best kinetic model fit including zero, first second orders, Higuchi, Weibull, and Korsmeyer–Peppas diffusion models, using KinetDS 3.0 (Aleksander Mendyk, GNU, Krakow, Poland. GPLv3 license, 2012) software.

#### 2.4.5. Solid State Characterizations and Compatibility Studies

Optimized NIC–NLC formula was lyophilized (Alpha 1–2 LD plus CHRIST, Osterode am Harz, Germany), for 12 h at −50 °C and subjected to the following studies:

##### Differential Scanning Calorimetry (DSC)

Samples (2–4 mg) of NLC–NLC, plain NIC, and physical mixture (PM) were separately transferred into an aluminum pan of differential scanning calorimeter (Perkin–Elmer DSC4, Boston, Massachusetts, USA), and continuously purged by a constant rate of 10 °C/min over a temperature range of (30–300 °C) against a blank under nitrogen gas, and thermograms were compared.

##### Infrared Spectroscopy (IR)

To test for any possible interactions between formula components, samples (2–4 mg) of NIC–NLC formulas and plain NIC were scanned by IR spectrophotometer (Shimadzu IR-435, Kyoto, Japan) in the range of 4000–500 cm^−1^ after compression with dry potassium bromide. The IR spectra were analyzed.

##### X-ray Diffraction (XRD)

Diffraction patterns of optimized NIC–NLC formula and plain NIC were studied using X-ray diffractometer (XRD-6000, Shimadzu, Japan) with Cu tube nodes. Samples were measured at a voltage of 40 kV, current of 40 mA, and 0.04 steps value. Data were presented on the diffractograms at 2– theta scattering angle range of 5–60° and scanning rate of 0.4 degrees per step. 

#### 2.4.6. pH Measurement

The pH of optimized NIC–NLC formula (1 mL) was measured after suitable dilution with double distilled water using a calibrated pH meter (SevenExcellence pH/mV meter; Mettler Toledo, Columbus, OH, USA). An average of three readings were recorded.

### 2.5. Ex Vivo Nasal Permeation Studies 

Permeation ability of the prepared NIC–NLC in comparison to NIC–Sol through nasal mucosal membrane was studied on sheep nasal mucosa (Rahmani, 7 months age, 30 Kg) using modified Franz diffusion cell. The intact membrane was carefully separated, cleaned, and stored in PBS (pH 6.4) at 4 °C until use [66].

For drug permeation study, excised membrane of suitable area (10 mm diameter, 0.2 ± 0.06 mm mucosal membrane thickness) was mounted on a diffusion cell between a donner compartment packed with a suitable volume of tested solution facing the mucosal side of the membrane and acceptor chambers containing SNF (pH 6.4). The cell top was securely sealed to avoid any leak. 

Permeation process through the membrane was allowed for 24 h under continuous stirring at 37 ± 0.5 °C. Aliquots (1 mL) were withdrawn from the acceptor compartment at specified time intervals and replaced with fresh SNF. The permeated drug in each sample was assayed by suitable HPLC. The permeation profile was constructed to calculate the steady state flux (Jss, µg/cm^2^ h), and the apparent permeability coefficient (Papp) was calculated using the following equation.

(3) $$Papp=\frac{\Delta Q}{\Delta T}\left(\frac{1}{ACo}\right)$$

where Δ*Q*/Δ*T* is the steady-state flux, *C_0_* is the initial concentration in the donor chamber (µg/mL), and A is the surface area of the mucosal membrane layer (cm^2^) [67]. The data were recorded as the means ± SD of six determinations. To control deviation in membrane thickness due to the permeation process, thickness was remeasured after completion of the experiment, if significant change (more than 25%) was observed, the results were discarded.

### 2.6. In Vivo Bioavailability and Brain Distribution Studies

#### 2.6.1. Study Design

The protocol of the study and experiments on animals were reviewed and approved by the research ethics committee for experimental and clinical studies at the Faculty of Pharmacy Beni-Suef University, Egypt.

The clinical protocol and information regarding nicergoline brain uptake was discussed with healthy adult, male Wistar rats (200–250 g, *n* = 24). Animals were housed in polypropylene cages under standardized temperature and humidity conditions with free access to water ad libitum. Rats were randomly divided into four groups: Group A and B received IN-optimized NIC–NLC formula and NIC–SOL, respectively. Group C and D received IV-optimized NIC–NLC formula and NIC–SOL, respectively, all animals received the drug in a suitable calculated dose for rat [68]. For IN administration, the dose was administered using a 0.1 mm polyethylene tube attached to a Hamilton syringe under anesthesia (pentobarbital sodium, 40 mg/kg, intra peritoneal), and the IV dose was administered through the tail vein [69]. At the specified time intervals, animals were sacrificed, and blood samples were collected in heparinized tubes and immediately centrifugated (5000 rpm for 10 minutes) to separate plasma which was then kept at –20 °C until analysis. Brains were separated, washed carefully with saline, weighed, and homogenized in PBS (pH 7.4). Homogenate samples were then centrifugated (5000 rpm for 15 min, 4 °C) and supernatant was collected and stored at –20 °C until analysis.

#### 2.6.2. Analysis of Samples and Drug Determination

The NIC concentration in the collected samples was assayed using a validated, sensitive, fast, and high-performance liquid chromatographic (HPLC) method [70] briefly described in the follow.

##### Chromatographic Conditions:

Mobile phase composed of (15:85 *v/v*) acetonitrile: Ammonium acetate buffer (0.1 mol/L, pH 6) was injected into the liquid chromatography unit equipped by Diamonsil ODS-C18 column (150 mm × 4.6 mm, 5 µm) at a flow rate of 1 mL/min at 30 °C. Quantitation was achieved using tizanidine hydrochloride as an internal standard on a UV–Vis spectrophotometric detector (SPD-10A) operated at a 224 nm detection wavelength.

##### Sample Preparation:

In 5 mL glass tube, aliquot samples (500 μL) of thawed test samples, 20 μL (1 μg/mL) internal standard (50% methanolic solution), sodium fluoride (10 mg), and 0.2 mL (5 Mol/L) NaOH were thoroughly mixed and vortexed for one minute. The mixture was then extracted with diethyl ether (2.5 mL) by vortex for 10 min followed by centrifugation (3500 rpm for 5 min). Supernatant organic layer was separated to another clean glass tube dried by evaporation at 40 °C until complete dryness. Residue was reconstituted in (70 μL) mobile phase, vortexed, and centrifugated (3000 rpm for 5 min). Finally, a 20 μL aliquot supernatant sample was directly injected into the HPLC system using the autosampler. The NIC concentration was calculated with reference to data from constructed calibration curves in plasma and brain homogenate extract.

##### Pharmacokinetics Calculations:

Plasma concentration–time and brain concentration–time curves were constructed and the pharmacokinetic parameters (namely, Cmax (μg/mL), Tmax (hr), AUC_0–12_ and AUC_0–∞_ (μg.hr/mL), Kel (h^−1^), and t_1/2_ (h)) for each rat following IN and IV administration were calculated and manipulated with the noncompartmental model using WinNonlin Professional 4.0.1 software (Pharsight corp., Cary, NC, USA).

For assessment of brain-targeting efficiency of tested formulations, the brain-targeting efficiency percentage (BTE%) that describes brain exposure to the drug after IN administration in comparison to IV administration data was calculated according to the following equation:

(4) $$\mathrm{BTE}\%=\left(\frac{\mathrm{BIN}/\mathrm{PIN}}{\mathrm{BIV}/\mathrm{PIV}}\right)\;\times \;100$$

where, BIN and PIN are the AUC_0-inf_ in brain homogenate (B) and plasma (P), respectively, after IN administration of optimized formulation and drug solution, while BIV and PIV are the AUC_0-inf_ in brain homogenate (B) and plasma (P), respectively, after IV administration. The BTE% of values range from 0 to +α. Efficient brain targeting after IN administration compared to IV is indicated by values above 100% [71].

The direct transport percentage (DTP%) that describes the fraction of the drug reached directly to the brain from the nose following IN dose versus the total drug amount reaching the brain after IN delivery. It was calculated using the following equation:

(5) $$\mathrm{DPT}\%=\left(\frac{\mathrm{BIN}-\mathrm{Bx}}{\mathrm{BIN}}\right)\times \;100$$

(6) $$\mathrm{While}{\;}_{\;}\mathrm{Bx}=\left(\;\frac{\mathrm{BIV}}{\mathrm{BIN}}\right)\;\times \;\mathrm{PIN}$$

Positive DTP% values up to 100% indicate a great extent of direct nasal drug transport to the brain, while DTP% value less than or equal to 0 indicate that the drug reaches the brain mainly through systemic circulation following IV administration [71].

##### Statistical Analysis of Pharmacokinetic Data

Brain and plasma pharmacokinetic parameters were subjected to statistical post hoc one-way analysis of variance ANOVA test (Tukey mode) at *p*-value > 0.05 (IBM-SPSS, Inc., Chicago, IL, USA).

### 2.7. Stability Study 

A stability of optimized NIC–NLC formula was studied by storage at room temperature (25 °C) and refrigerating conditions (4 °C) for one month. Samples (10 mL) were stored in a closed glass vial. The LC, ZP, PS, and PDI of formula were measured once at the end of the storage period and the results were compared.

## 3. Results and Discussion

Quality should not be tested into products but should be built in; this statement describes the concept of “quality by design” [72].

Statistical experimental design gives the advantage of determination and assessment of different factors that affect and control a predefined response reaction and determine their appropriate levels through minimum experimental planning [73].

Being simple with relatively few experimental runs in comparison to other similar designs, Box-Behnken, a response surface methodology technique, [74] was selected and applied in our study.

A matrix design of seventeen runs (including five center points) represents the NIC–NLC that was constructed. Formulations were prepared and subjected to analysis, and the observed response values were recorded (Table 2). Significant changes in response values when the factor and/or its level changed indicates the suitability of the independent factors selection due to the high dependency of formulation characteristics (responses) on their values and levels.

In polynomial model equation, A, B, and C represent the independent variables, while (AB, AC, and BC) and (A^2^, B^2^, and C^2^) signify the interaction and quadratic terms, respectively [75]. Reproducibility of experimental results is affected with applied controllable factors, while variability results from uncontrollable factors, which is described as noise and measured in terms of adequate precision. Values greater than four are usually desirable. The results in Table 3 indicate a low noise signal with an insignificant effect on the collected results and support the suggested model for describing the proposed experimental design [74].

Fitting of the collected results to different polynomial mathematical model equations suggested the linear, quadratic, and 2FI models to describe the effect of selected independent variables on PS, ZP, and EE of the NIC–NLC formulation, respectively, where higher proximity between predicted and adjusted coefficients (<0.2) was observed with significant statistical model term at the selected probability level and the least SD values (Table 3).

Further validation of derived mathematical model equations using ANOVA, as shown in Table 4, indicated significant F-values for the proposed statistical models with adequate signal and a minimum noise chance of only 0.01%, where the recorded probability was less than or equal to 0.0001 for the three responses. Insignificant value of lack of fit for the three proposed models indicated best fit with least error (noise) mainly due to the good selection of variables and ensured strength of applied experimental design [76]. These results agreed with the calculated adequate precision values and ensure the suitability of the suggested model equations to navigate the design space.

For further investigation of the effect of selected independent factors on the desired response, 3D response surface plots were constructed, where factorial interactive binary effects on definite response can be studied at a constant level of the third factor. To track the performance of any response when only one factor changes within the proposed constraint range while the other two factors kept constant, perturbation plots were also constructed. They also allow comparative study of all factors at any selected point in the design space [64].

### 3.1. Effect on Particle Size

**Figure 1:**
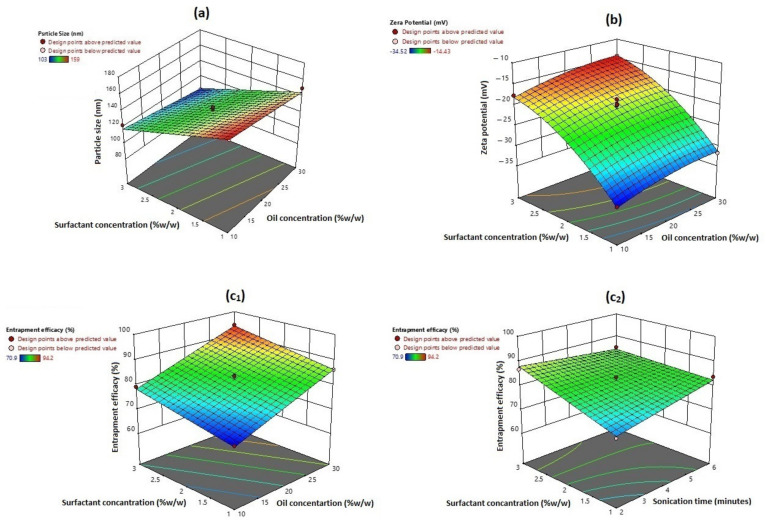
3D surface plot for the effect of oil concentration (%), surfactant concentration (%), and sonication time on (**a**) particle size, (**b**) zeta potential, and (**c_1_**,**c_2_**) entrapment efficacy (%). Oil concentration and surfactant concentration had a significant effect on particle size, zeta potential, and entrapment efficacy, while sonication time also showed an interactive positive effect on entrapment efficacy.

Particle size of colloidal lipoid systems is a complicated process as it is affected by types of lipid and/or surfactant used, their amounts and relative ratios, also can be controlled by applied formulation conditions of homogenization and/or sonication speed and time [77]. The results presented in Table 2 and illustrated in Figure 1A show that the PS was in the range of 103.46 nm (F10)–159.78 nm (F6). The effect of A, B, and C on particle size was quantitively described by the following polynomial regression equations for linear model:

PS = 131.76 − 7.00 A − 20.50 B − 1.50 C(7)

This coded equation can be applied to predict particle size at any level of the specified independent factor, where the positive value of a factor coefficient designates a proportional effect on response and a negative value indicates an inverse relationship between this factor and tested response. Only model terms of P > F < 0.05 are considered significant. 

According to the ANOVA results in Table 4, only the model terms A and B, significantly affect the prepared NIC–NLC system particle size. The negative sign of coefficients for both factors reflects their antagonistic effect on particle size. Results showed that increasing the liquid lipid (sesame oil) content of the total lipid (A) result in smaller particle size, where F5, F10, F15, and F17 (A = 30%) showed significant smaller particle size than F12, F14, F6, and F13 (A = 10%) respectively at constant values for B and C. 

In colloidal lipoidal systems, increasing the lipid concentration results in the formation of larger particles due to the higher tendency of lipids to coalesce with the concentration increase that results in increased viscosity and consistency of the formula mixture with a subsequent increase in surface tension [64]. This effect was dramatically decreased when liquid lipids were added to the mixture, and this could be explained by the negative effect of liquid lipids on mixture consistency and decreasing surface tension. In addition, the presence of oil prevents the formation of perfect lipid crystal, as the excess oil residue that is not involved in the lipid crystallization process hinders further crystallization of solid lipids which results in the formation of smaller particles [78].

**Figure 2:**
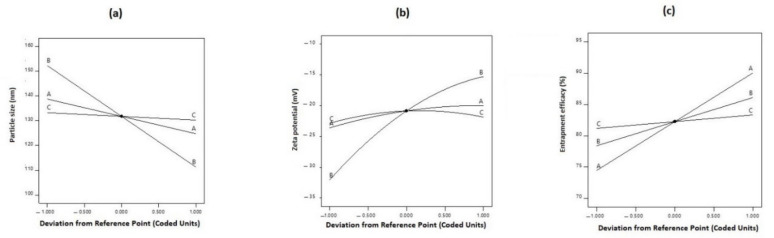
Perturbation plot of effect of (A) oil concentration (%), (B) surfactant concentration (%), and (C) sonication time (min) on (**a**) particle size, (**b**) zeta potential, and (**c**) entrapment efficacy (%). Surfactant concentration (B) had a prominent main linear effect on particle size and quadratic nonlinear effect on zeta potential, while oil percentage (A) had a prominent main linear effect on entrapment efficacy (%).

Increasing the SAA (B) concentration within the formula mixture results in smaller particle size, where F1, F4, F10, and F14 (B = 3%) showed significant smaller particle sizes than F9, F8, F15, and F16 (B = 1%), respectively, when B and C were kept constant. This effect was directly related to the lowering of the surface tension of the liquefied melted lipid mixture and increasing the rate of subdivision of lipoidal droplets in the formed primary emulsion that resulted in smaller particles [79]. High shearing processing conditions applied during formula preparation led to an increase in the surface free energy (SFE) of the formula mixture; this decreased the stability of the formed lipoid droplets and caused coalescence of the droplets with an increase in particle size. Lowering of the surface tension by SAA addition causes a significant decrease in SFE energy and prevents the undesired reaggregation process and production of smaller particles [64]. This describes the smallest particle size attained at a higher concentration of liquid lipid concentration and SAA applied (F10). In this work, the maximum concentration of SAA applied was 3%, as high SAA concentration my affect formula stability during storage due to the fact of its effect on zeta potential. Moreover, a high SAA concentration may increase the cytotoxicity of the formula and affects drug release [80]. Figure 2A illustrates the perturbation plot of the effect of different factors on PS, where the SAA concentration had the major effect followed by oil percentage, while the ST almost had no effect on the PS. The linearity of the curve signifies the main non-interactive effect on particle size.

The PDI is the criterion of the PS distribution homogeneity. It is calculated based on the measured standard deviation and the mean particle size of specified dispersed system. Homogenous, monodispersed systems are described by small values of PDI indicating a narrow PS distribution range with higher stability [81]. The results in Table 2 show that all prepared NIC–NLC systems had PDI values in the range from 0.128 to 0.305, indicating a good size distribution and homogeneity of the prepared NLC systems.

### 3.2. Effect on Zeta Potential

Zeta potential is an electro-kinetic potential that governs the stability of colloidal systems [81]. Particle size reduction during the preparation process increases the surface area and SFE; this results in a thermodynamically unstable system and nanoparticles tend to coalesce and reaggregate. Repulsion between adjacent nanoparticles prevents reaggregation and stabilization of the dispersed system. Zeta potential must be high enough (>±30 mV ) to maintain stability during storage. [77]. Despite cationic, nanoparticles show higher affinity to nasal mucosa due to the electrostatic interactions with negatively charged nasal epithelial cell membranes with enhanced cellular uptake; they cause abrupt toxic effects on the BBB due to the disruption of the microvasculature endothelium within one hour [82]. In contrast, neutral and anionic nanoparticles of moderate charge did not affect the BBB’s integrity and showed significant rapid increase in brain permeability due to the interaction with low-density lipoprotein receptor on the BBB and bulk flow transport through the extra neuronal pathway for brain distribution. These data refute the initial hypothesis that negatively charged nanoparticles would be repelled at the BBB [83].

For that, dicetyl phosphate (0.03% *w/v* based on trial experiment) was added as a charge modifier to induce controlled moderate negative zeta potential on the nanoparticle’s surface.

Zeta potential (R_2_) values for the prepared NIC–NLCs were in the range from −14.43 mV (F10) to −34.52 mV (F6). The interaction effects of selected formulation factors on zeta potential values were quantitively expressed by the following polynomial regression equation for quadratic model:

ZP = − 20.85 +1.82 A + 8.39 B +0.4987 C +0.1325 AB − 0.0300 AC + 0.3625 BC − 0.9350 A^2^ +2.82 B^2^ − 1.51 C^2^ (8)

According to the ANOVA results, only model terms A, B, and B^2^ were significant with *p*-values less than 5% (Table 4). Despite the positive sign of the coefficient indicating a synergistic effect on a specified response, here the reverse was considered due to the negative value of the measured zeta potential. Liquid lipid percentage showed the main (A) antagonistic effect on the zeta potential. In other words, the higher the liquid lipid concentration, the smaller the negative charge density on the particle surface, and this could be correlated to the negative effect of liquid lipid percentage on particle size; smaller particles can only accommodate smaller charge density due to the smaller surface area [64]. The concentration of added surfactant also showed antagonistic main (B) and quadratic (B^2^) effects of the zeta potential. Figure 1B illustrates the statistical surface plot that describes this effect: zeta potential values showed a significant decrease for F14, F4, F1, and F10 prepared with higher SAA concentration (B = 3%) when compared to F6, F8, F9, and F15, respectively, that were prepared with a lower SAA concentration (B = 1%) when A and C were held constant. Further justification of results based on constructed perturbation plot, as shown in Figure 2B, and values of factor coefficients in the polynomial equation showed that the main effect of SAA concentration had a prominent impact on ZP with a higher value (8.39) in comparison to other significant coefficients in a polynomial equation, while a nonlinear curve signified the quadratic effect.

### 3.3. Effects on Entrapment Efficiency

The prepared NIC–NLCs showed high EE% in all formulations ranging from 70.9% (F6) to 94.2% (F10) at different levels for the three selected formulation factors (Table 2). The two-factor interaction (2FI) model showed the best fit for describing the effect of different independent factors on EE according to the following equation:

Entrapment efficacy = + 82.26 + 7.79 A + 3.85 B + 1.07 C − 0.0675 AB + 0.2700 AC + 2.93 BC
(9)

According to ANOVA results, only the model terms A, B, and BC showed a significant effect on EE with P > F < 0.05 (Table 4). Liquid lipid percentage (A) and SAA concentration (B) showed positive synergistic main effects on EE%. In other words, the EE% increased when each factor increased independently. The effect of oil percentage on EE% was prominent with a higher coefficient value (7.79). The results presented in Table 2 and illustrated in Figure 1c1 showed a significant increase in EE% for NIC–NLC formulations F6, F12, F13, and F14 when compared with formulations F5, F10, F15, and F17, respectively, that were prepared at the same levels for other factors when oil percentage increased from 10% to 30%. Incorporation of oil hinders recrystallization of solid lipids after cooling and causes formation of imperfect lipid crystals that may result in amorphous lipid mass; this results in a higher accumulation of the drug within the lipid phase. Oil addition also reduces viscosity of the lipid mixture that increases the drug dissolution rate within and results in higher drug entrapment [84]. The second significant factor that affects the EE% is the SAA concentration. The NLC formulations F6, F8, F9, and F15 showed higher EE% for NIC when compared to NLC formulations F1, F4, F10, and F14, respectively, when the SAA concentration increased from 1% to 3% when other factors were kept constant to confirm the main positive effect of SAA concentration on EE%. This effect could be correlated to the solubilizing action of SAA at higher concentration and accumulation of Tween 80 (SAA) interfacial film on the globular surface allowing for the accommodation of additional drug [58,81].

The only significant interactive effect for the different factors on EE% is the simultaneous change in both SAA and sonication time (BC). The positive value of the factor coefficient indicates proportional relation while its smaller value (2.39), in comparison to other factor coefficients, reflects a lesser impact of this factor. 

Tracking of this interactive effect on the response surface plots, Figure 1C_2_, shows that, changing ST away from SAA concentration had no significant effect on EE%. This could be correlated to the high viscosity of the lipid mixture that hinders the drug solubility, increasing EE% at higher ST (C = 6) from 74.6% (A = 10%, F13) to 91.18% (A = 30%, F17) when SAA concentration was kept constant (B = 2) supports this explanation. On the other hand, simultaneous increase in both SAA and ST resulted in higher NIC EE% when (A) was kept constant at all three levels (10−30%). The EE% increased from 73.6% (F9) to 85.1% (F4) when both factors increased simultaneously from their lower to higher values at a constant oil percentage (20%). These results also confirm the proposed explanation, where increasing the activation energy of drug molecules within the lipoidal mixture under the effect of sonication action overcomes the viscosity effect and augments the solubilizing action of SAA and causes a significant increase in drug EE%. The perturbation plot, as shown in Figure 2C, confirms the priority of oil percentage concentration to control EE%, while the linearity of the curve signifies its main effect.

### 3.4. Optimization and Validation of the Collected Data

Results in Table 5 show acceptable agreement between the observed and predicted numerical data for the three responses within the proposed constraints of the tested independent formulation factors according to the proposed design matrix and calculated desirability. These results indicate validity of the selected optimization method and suitability of the derived regression model equations for prediction of PS, ZP and EE% of NIC–NLCs at the tested formulation variable levels.

### 3.5. NIC Release Studies

**Figure 3:**
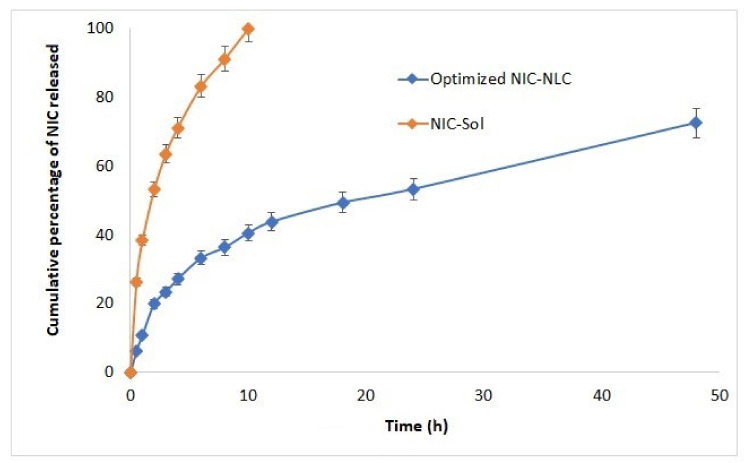
Release profiles of NIC from optimized NIC–NLC formulations (average release data of the three optimized NIC_NLC formulations was illustrated) in comparison to plain NIC in simulated nasal fluid (pH 6.4).

The release profile of NIC from optimized NLC formulations (F1–F3) in simulated nasal fluid was studied and the average release data were illustrated in Figure 3. Nicergoline showed retarded slow-release pattern from the prepared NLC formulations over 48 h and reached approximately 72% of the dose, while the unformulated drug reached 71% after 4 h only and completely dissolved within 10 h. This effect is mainly related to the lipophilic nature of the drug that slowing its partitioning out of the lipid core of the NLC systems [58]. Also, absence of this barrier for drug diffusion at the release membrane surface allows better and faster drug availability in dissolution medium in unformulated form [54]. Further analysis of the release data show that, the release profile of NIC from NLCs formulations revealed a biphasic release pattern with an early fast release within the first two hours (reached approximately 20%), followed by a delayed slow-release pattern over 48 hrs. Biphasic release patterns are usually expected in matrix-based formulation systems, including NLC, where matrix biodegradation and diffusion in addition to erosion of the surface layer are the main mechanisms governing drug release [64]. Initial fast release of the drug occurs due to the dissolution of the adsorbed drug on particulate surface and localized in the outer most layer of the lipoidal matrix. Lack of homogeneity within the internal phase of NLCs due to the difference in melting points between solid and liquid lipids causes escaping of oil from the core and accumulation on the outer most layers. Low viscosity of liquid lipids and better drug solubility allow easier diffusion of the drug and faster erosion of the matrix surface [54,85]. In addition, the tendency of Tween 80 to precipitate onto the globular surface also supports this initial burst release [58]. Over time, slower drug release occurs from the inner core in which the solid lipid is almost homogenous and closely packed; this allows only slow and controlled penetration of the dissolution medium into the deeper lipid layers and drug dissolution. Erosion of the outermost layers also raises the viscosity of the stagnant layers and delays the diffusion process with negative effects on drug release [77,86].

Fitting of release data to different kinetic models suggested the Korsemeyer–Peppas model with the best fit based on the higher correlation coefficient (R^2^ = 0.9850) to describe the release mechanism of NIC from optimized NLC formulas. These results reflect multistep complicated release pattens, including diffusion, expansion and/or swelling followed by dissolution. These data are completely consistent with the nonperfect nature of the lipid matrix due to the presence of oil with a subsequent effect on drug distribution that is optimally described by Korsemeyer–Peppas rather than the Weibull kinetic model, which usually reflects uniform drug destitution within a perfect solid lipid matrix. Further analysis of data based on the calculated mathematical model equation showed (A) values greater than 0.89 with super case-II release characteristics due to the matrix erosion–disentanglement prominent release behavior [64].

### 3.6. Thermal (DSC) Analysis 

**Figure 4:**
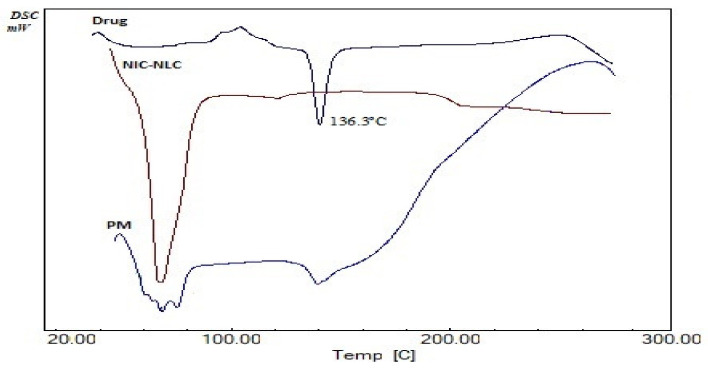
DSC thermograms of NIC (drug) compared to optimized NIC–NLC formula and physical mixture (PM) of formula components.

A DSC analysis is a usually applied as a simple and reliable tool to investigate drug purity and compatibility within a specified formulation mixture. It also provides data on drug crystallinity and distribution characteristics within a certain matrix [87]. Figure 4 shows the DSC thermogram of plain NIC in comparison to the optimized NIC–NLC formula and formula physical mixture. NIC showed a sharp endothermic melting peak at 136.3 °C in consistent with the data in the literature [88], signifying the purity of the drug. The drug peak was retained in physical mixture as an indicator for drug crystallinity and compatibility of the formula mixture. The DSC thermogram of the optimized NIC–NLC formula did not show the drug melting peak without appearance of any other new peaks, this indicates changing the drug to the amorphous state. This could be correlated to rapid quenching of lipids during preparation of NLCs and the presence of SAA that inhibit drug crystallization [54]. Loss of drug crystallinity also confirmed uniformity of drug distribution within the lipid matrix. 

### 3.7. Infrared Spectroapy Stuies

**Figure 5:**
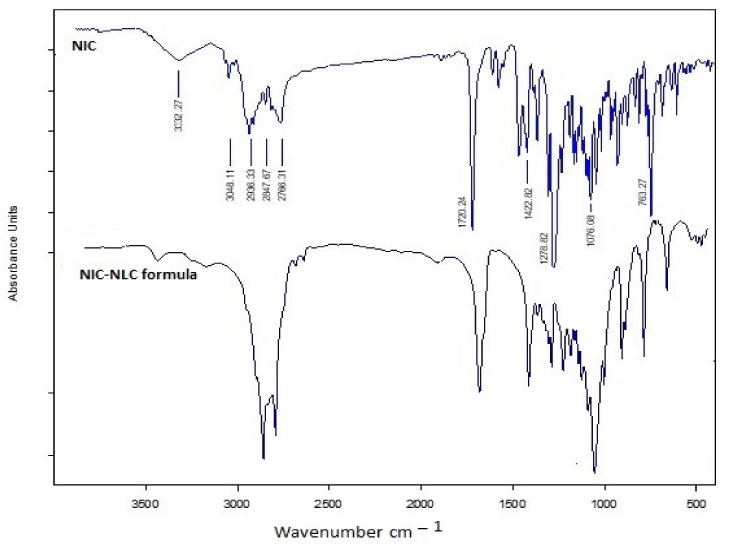
IR Spectra of NIC in comparison to optimized NIC–NLC formula.

Figure 5 shows the IR spectra of NIC main functional characteristic groups at 3332.27 cm^−1^ (primary N–H), 1720.24 cm^−1^ (C=O stretching), 1422.82 cm^−1^ (C–H stretching), 1270.82 cm^−1^ (C–N stretching), 1078.06 cm^−1^ (aliphatic C–C), and 763.27 cm^−1^ (Ortho distribution) in consistent with the data in the literature [89]. These peaks were all retained in IR spectra of the optimized NIC–NLC formula to confirm the compatibility of the optimized NIC–NLC formula mixture and eliminates the possibility of any chemical interaction.

### 3.8. XRD Analysis

**Figure 6:**
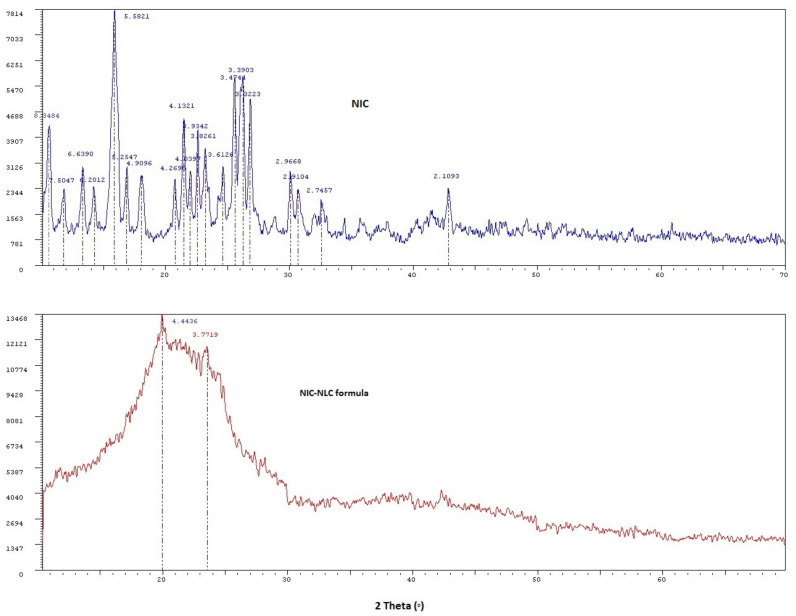
XRD diffractograms of NIC compared to the optimized NIC–NLC formula.

The XRD analysis was applied to identify the crystallinity of solids, as a crystalline solid XRD pattern is unique, and my may be considered as a fingerprint [87]. Figure 6 shows the XRD pattern of NIC with sharp intense peaks at 2 theta scattered angels of 10.58, 11.78, 13.33, 14.27, 15.86, 16.87, 18.06, 20.79, 21.48, 21.99, 22.59, 23.22, 24.63, 25.62, 26.27, 26.81, 30.11, 30.69, 32.59, and 42.84° reflecting high crystallinity of the plain drug. These peaks almost completely disappeared in the XRD pattern of the optimized NIC–NLC formula sample, indicating loss of drug crystallinity due to the change in the amorphous state and/or complete solubility within the lipid matrix. These results are in accordance with DSC results to confirm efficient drug dispersion within the lipid matrix of NLC systems.

### 3.9. pH Measurement

To avoid irritation of nasal mucosa and prevent further complications on nasal ciliary beats and olfactory neurons, the pH of human nasal mucosa must be retained in the normal range 5.5−6.5) [90]. The pH of the optimized NIC–NLC formula was measured and was 5.8.; these results ensure the biocompatibility of the prepared NIC–NLC intranasal delivery system.

Based on the previous evaluation data, the optimized NIC–NLC formula (F1), composed of 30% sesame oil (of total lipid), 3% Tween 80 and prepared by sonication for 4.17 min, was prepared and subjected to further evaluation Ex vivo permeation and in vivo bioavailability studies.

### 3.10. Ex Vivo Permeation Studies

**Figure 7:**
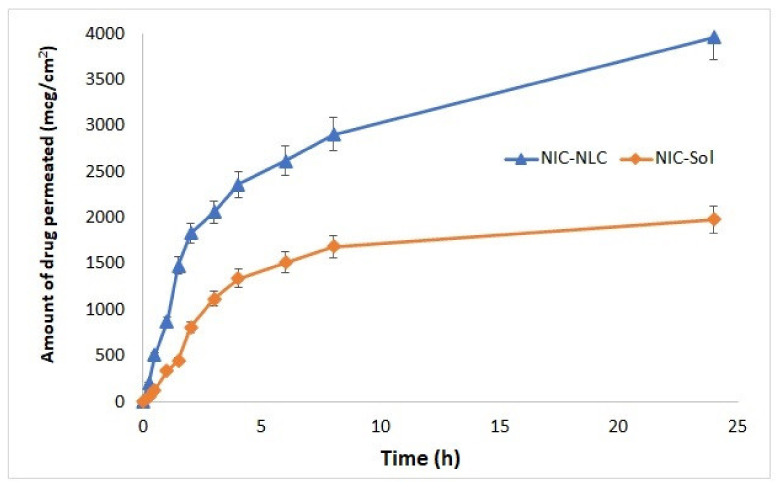
Ex vivo permeation profiles of the optimized NIC–NLC formula compared to NIC–SOL through sheep nasal mucosal membrane.

To study the diffusion properties of the prepared NLC delivery systems, ex vivo permeation through sheep nasal mucosa was investigated for the optimized NIC–NLC formula in comparison to NIC–SOL, and permeation profiles showing the cumulative amount of NIC permeated per unit area (µg/cm^2^) against time (h) over 24 h are illustrated in Figure 7. The NIC flux (Jss), which was calculated as the slope of terminal linear part of permeation profile curve, was 97.77 µg/cm^2^ h and 42.47 µg/cm^2^ h for NIC–NLC and NIC–SOL, respectively, with an enhancement ratio of 2.3. Also, the calculated apparent permeability (P_app_) which designates the flux in terms of the rate of drug accumulation in the acceptor compartment of the Franz cell per unit surface area of diffusion membrane was 19.55 cm h^−1^ and 8.49 cm h^−1^, respectively, indicating significant enhancement of the amount of NIC permeated through the nasal membrane from the prepared NIC–NLCs. 

These results could be correlated to the physical characteristics and composition of NLC systems. Regarding the physical characteristics, the nano-size feature was the main reason for the permeability increase, because particles of 1 to 500 nm size can squeeze within the aqueous, non-viscus pores of the mucin network; this directly results in higher mucosal permeation and internalization within the mucosal cells [57]. A moderate negative ZP of the nanoparticles may also play a significant role in preventing adherence of the NIC–NLC onto to the negatively charged membrane, which may retard the permeation process. The NLC system’s composition was another important cause for the permeability increase through the nasal membrane, as the lipophilicity nature of the nanoparticles favors their transfer across the membrane through passive diffusion. In addition, the presence of Tween 80 as SAA played an important role as a permeation enhancer; the hydroxyl group can interact with the anionic oxygen of phospholipid membrane polar heads and cause distortion of the intercellular lipid pack of membrane, resulting in increased membrane porosity and enhanced permeability [91].

### 3.11. In Vivo Bioavailability and Brain Distribution Studies

**Figure 8:**
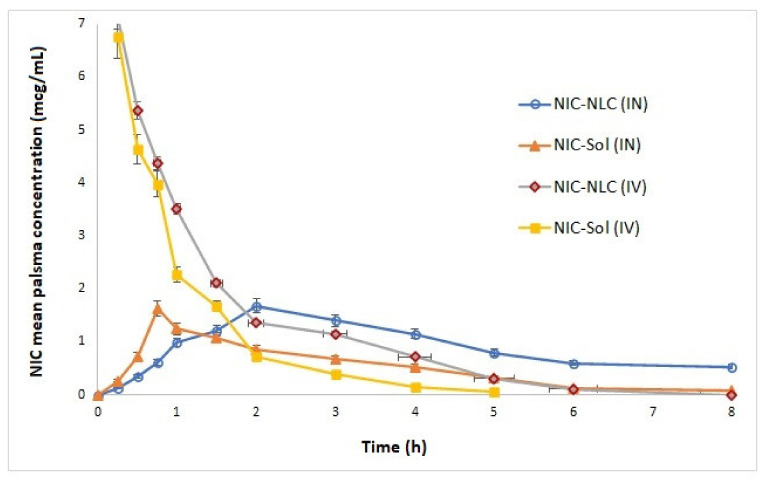
Mean plasma concentration–time profiles of optimized NIC–NLC compared to NIC–SOL following IN and IV administrations to Wister rats.

**Figure 9:**
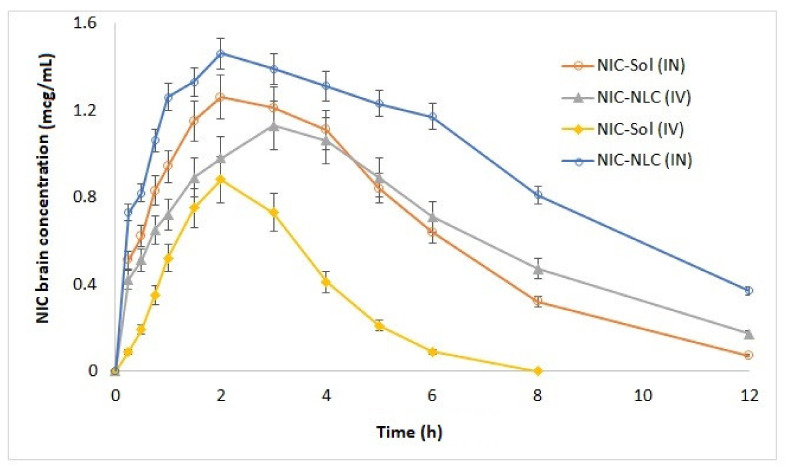
Mean brain homogenate concentration–time profiles of optimized NIC–NLC compared to NIC–SOL following IN and IV administrations to Wister rats.

To investigate the effect of NLC delivery system on brain distribution and bioavailability of NIC, the pharmacokinetics of optimized NIC–NLC formula were studied in both the plasma and brain tissue following IV and IN administrations in comparison to NIC–SOL in male Wistar rats. Pharmacokinetic parameters are summarized in Table 6, and biodistribution profiles are illustrated in Figure 8 and Figure 9.

The optimized NIC–NLC formula showed a significantly higher concentration (Cmax) following IN administration in plasma (1.68 µg/mL) and brain (1.46 µg/mL) when compared to NIC–SOL (1.08 µg/mL and 0.75 µg/mL), respectively, without any effect on Tmax, which was kept constant at 2 h in all cases. Regarding bioavailability, the optimized NIC–NLC formula also showed significant improvement of the extent of drug absorption with relative bioavailability of 167.79% and 457.3% in plasma and brain, respectively, in comparison to NIC–SOL. These results agree with that published by K. Jain et al. [54], who reported a significant increase in bioavailability of artemether NLC following IN administration when compared to drug solution. These results could be correlated to the small particle size and lipophilic nature of NLCs that allow for better permeation into nasal mucosa as previously discussed in ex vivo studies.

The tight junctions between the olfactory bulb and cerebrospinal fluid (CSF) represents a considerable pathway for nasal drug delivery to the CSF when the drug and/or formula has the ability to penetrate both nasal epithelia and the arachnoid membrane [92]. The presence of Tween 80, with its solubilizing ability and its effect on distortion of lipoidal membrane integrity, allows for better transcellular transport through olfactory neurons to the brain by several endocytic pathways of neuronal and/or sustentacular cells present in the olfactory membrane and reach lamina propria instead of paracellular pathway, the process is size dependent occurs optimally with PS range of 100 nm and can be observed with a maximum of 200 nm [93,94]. Further interpretation for PS’ effect on enhancement of permeation to the brain tissue was discussed by Bourganis et al. [95], who proved a relationship existed between PS of colloidal formulations and diameter of the olfactory axons, where nanoparticles with a PS smaller than 500 nm can easily permeate and show better cellular uptake and intracellular transport via olfactory neural pathway to the brain. Significant increases in bioavailability could also be explained by the mucoadhesive nature of NLCs that overcomes the mucociliary clearance action of nasal mucosa [91]. Another reason to be considered is the ability of NLC to protect the drug from enzymatic degradation within the nasal mucus [54]. Presence of NIC in plasma with considerable concentrations following IN administration of NLC formula and drug solution was acceptable as systemic absorption is expected through the respiratory epithelium due to the high vascularity nature [96]. Further investigation of pharmacokinetics data showed that NIC–NLC formula had longer plasma half-life (3.17 h) in comparison to NIC–SOL (1.45 h) following IN administration, the extended circulation time of NIC–NLC formula allows exposure of a larger area of nasal mucosa to the drug for a longer time, which results in extended drug absorption and higher bioavailability [58].

To study the efficiency of the prepared NIC–NLC formula for brain targeting, BTE% was calculated, which numerically compares the brain’s exposure to NIC following IN and IV routes. The BTE% of the NIC–NLC formula was 187.3%, while the BTE% for NIC–SOL was 92.6%. Values higher than 100% indicating efficient brain targeting of the NLC formula. These results confirm the previously discussed pharmacokinetic data and reflect the positive effect of PS, lipophilicity, and formula composition on drug permeation through the olfactory pathway to the brain tissue. 

Following transcellular permeation of the olfactory membrane and when nanoparticles reach the lamina propria, they may be absorbed into systemic circulation, reach cervical lymph nodes, or extracellularly diffuse to cranial compartment through the perineural and perivascular spaces; these pathways are expected alone or in combination [97].

For that, the DTP% was calculated to explore the main pathway for drugs to reach the brain tissue following IN administration for both the NIC–NLC formula and the NIC–SOL in comparison to IV data. The DTP% for the NIC–NLC formula was 56.64% and for NIC–SOL it was 180.6%. The positive value of DTP% for the NLC formula designates the direct nose-to-brain as the main pathway for drug transfer to the brain rather than systemic absorption through the BBB; NIC–SOL mainly reached the brain through the blood with preferential absorption through the BBB rather than the olfactory pathway as indicated by the high negative value for DTP%. These results confirm the extended residence time of the NLC formula within nasal mucosa when compared to the drug solution.

Statistical analysis of collected pharmacokinetic data (Table 7, Table 8, Table 9 and Table 10) indicated significance of the calculated differences in extent of drug absorption in terms of Cmax, Kel, and t_1/2_ between optimized NIC–NLC formula and NIC–SOL following IN administration at the selected level of probability.

### 3.12. Stability Study 

Results of the stability data presented in Table 11 indicated minimum changes in physical properties of optimized NIC–NLC formula including LC, ZP, PS, and PDI. At room temperature (25 °C), there was a slight increase in PS (132.65 mm) and ZP (−19.45 mV), while LC showed a slight decrease (4.3%). Storage at refrigerating conditions (4 °C) showed non-considerable changes in LC, ZP, and PS; PDI was not affected at all storage conditions. Statistical analysis of stability data indicated insignificance of the recorded changes in physical properties and signifies stability of optimized NIC–NLC formula.

## 4. Conclusions

Cognitive function disorders, mainly dementia, due to the presence of cerebral vascular disorders and Alzheimer’s disease, have shown significant increases due to the advances in health care services and increased life spans of human. Moreover, significant changes in the BBB’s permeability with age were correlated to cerebral vascular diseases and elevated amyloid protein levels in the basal lamina and tight junctions of the BBB result in impairment of cognitive functions and dementia. In this work, nicergoline, a selective, potent α1-adrenergic receptor agonist resulting in the improvement of cerebral circulation, was formulated into an NLC intranasal delivery system using sesame oil, a powerful antioxidant with a protective effect to maintain the BBB’s integrity. The NIC–NLC systems were optimized for higher EE%, smaller PS, and optimum ZP using oil percentage, SAA concentration, and ST as independent variables using a three level, three factor Box–Behnken statistical design. According to the collected results, oil percentage and SSA concentration had significant effects on PS and ZP, while the three factors significantly affected EE% according to different mathematical models. The optimized NIC–NLC formula had 111.18 PS nm, −15.41 ZP, 95.11% EE%, and 4.6% loading capacity with 98.7% desirability and low prediction error percentage. The optimized NIC–NLC formula showed an extended slow-release profile and reached 72% within 48 h. The solid-state characterization studies using DSC, IR, and XRD indicated drug distribution within the lipid matrix in the amorphous form and good compatibility between drug and formula components. Ex vivo permeation studies through sheep nasal mucosal membrane showed a 2.3 enhancement ration in comparison to the drug solution based on calculated steady-state flux and permeability coefficient mainly due to the small PS, physical characteristics, and lipophilic nature of NLC delivery system. Finally, the in vivo bioavailability and brain distribution studies following IN administration in rats showed 4.57 fold increase in brain in comparison to NIC–SOL. The calculated BTE% and DTP% for IN optimized NIC–NLC were 187.3 and 56.6, respectively, indicating efficient direct nose-to-brain targeting properties. Based on these results, we can conclude that the prepared optimized NIC–NLC formula mainly transported to the brain directly after IN administration, which resulted in higher drug concentration and bioavailability in cranial tissue. The prepared NLC formula can be considered as a promising delivery system for direct nose-to-brain targeting of NIC with high levels and expected maximized therapeutic action.

## Figures and Tables

**Table 1:** NIC–NLC formulation variables with different levels in a Box–Behnken design.

Factor	Level		
	−1	0	1
Independent variables			
A: Oil: Total lipid (% *w*/*w*)	10	20	30
B: Surfactant concentration (% *w*/*w*)	1	2	3
C: Sonication time (minutes)	2	4	6
Dependent variables	Constraint	Importance	
R1: Particle size (nm)	Minimize	4	
R2: Zeta potential (mV)	−(15–20)	5	
R3: Entrapment efficacy (%)	Maximize	5	

**Table 2:** Composition matrix of the NIC–NLC formulations with observed responses.

Formula	A Oil (% *w/w*)	B Surfactant (% *w/w*)	C ST (min)	R1 PS ± SD (nm)	R2 ZP ± SD (mV)	R3 E.E ± SD (%)	PDI ± SD
F1	20	3	2	112 ± 3.2	−17.91 ± 1.2	86.7 ± 3.2	0.224 ± 0.02
F2	20	2	4	126 ± 4.1	−19.86 ± 2.3	80.6 ± 4.1	0.251 ± 0.02
F3	20	2	4	132 ± 3.8	−20.04 ± 0.7	81.3 ± 2.6	0.213 ± 0.04
F4	20	3	6	107 ± 2.7	−15.96 ± 0.9	85.1 ± 3.7	0.212 ± 0.04
F5	30	2	2	127 ± 3	−21.52 ± 1.2	90.6 ± 5.2	0.188 ± 0.05
F6	10	1	4	159 ± 5.1	−34.52 ± 1.7	70.9 ± 2.9	0.214 ± 0.03
F7	20	2	4	134 ± 4.6	−23.77 ± 2.3	81.9 ± 3.1	0.293 ± 0.04
F8	20	1	6	147 ± 3.9	−33.18 ± 3.1	83.7 ± 1.6	0.305 ± 0.02
F9	20	1	2	153 ± 3.9	−33.68 ± 1.6	73.6 ± 3.1	0.261 ± 0.01
F10	30	3	4	103 ± 2.7	−14.43 ± 0.8	94.2 ± 2.2	0.225 ± 0.05
F11	20	2	4	119 ± 2.2	−18.59 ± 1.5	80.08 ± 1.4	0.206 ± 0.04
F12	10	2	2	143 ± 5.7	−25.84 ± 2.1	75.1 ± 0.9	0.161 ± 0.03
F13	10	2	6	140 ± 5.3	−25.01 ± 1.3	74.6 ± 1.7	0.142 ± 0.05
F14	10	3	4	123 ± 3.1	−17.71 ± 0.7	79.2 ± 3.2	0.270 ± 0.03
F15	30	1	4	150 ± 6.2	−31.77 ± 4.1	86.17 ± 1.7	0.128 ± 0.01
F16	20	2	4	136 ± 4.1	−21.99 ± 1.1	83.56 ± 1.3	0.229 ± 0.02
F17	30	2	6	129 ± 2.9	−20.81 ± 2.2	91.18 ± 3.9	0.211 ± 0.03

ST: Sonication time, PS: particle size, ZP: zeta potential, EE: entrapment efficacy, SD: standard deviation, PDI: polydispersity index.

**Table 3:** Regression analysis results and ANOVA for different response surface models.

Formula	NIC–NLC Formulations						Remarks
	R Squared	Adjusted R Squared	Predicted R Squared	SD	CV%	Adequate Precision for ANOVA	
**R1 (Particle size)**							
Linear model	0.9180	0.8990	0.8797	5.09	3.86	22.2677	Suggested
2FI model	0.9269	0.8831	0.8383	5.48			
Quadratic model	0.9502	0.8861	0.8747	5.41			
Cubic model	0.9535	0.8139	-	6.91			Aliased
R2 (Zeta potential)							
Linear model	0.8953	0.8711	0.8498	2.31			
2FI model	0.8962	0.8339	0.7534	2.26			
Quadratic model	0.9734	0.9391	0.9355	1.59	6.8	16.7850	Suggested
Cubic model	0.9749	0.8998	-	2.04			Aliased
R3 (EE)							
Linear model	0.9158	0.8963	0.8385	2.08			
2FI model	0.9673	0.9476	0.8933	1.48	1.8	24.5089	Suggested
Quadratic model	0.9747	0.9422	0.7518	1.56			
Cubic model	0.9892	0.9566	-	1.35			Aliased

**Table 4:** Coefficients of different formulation variables according to the model of best fit.

Source	R1 (Particle size) Linear Model		R2 (Zeta Potential) Quadratic Model		R3 (E.E.%) 2FI Model	
	F-Values	*p*-Values	F-Values	*p*-Values	F-Values	*p*-Values
Model	48.49	<0.0001 *	28.41	0.0001 *	49.26	<0.0001 *
A	15.12	0.0019 *	10.52	0.0142 *	221.49	<0.0001 *
B	129.67	<0.0001 *	223.90	<0.0001 *	54.15	<0.0001 *
C	0.6942	0.4198	0.7907	0.4034	4.19	0.0677
AB			0.0279	0.8721	0.0083	0.9292
AC			0.0014	0.9709	0.1329	0.7230
BC			0.2089	0.6615	15.60	0.0027 *
A^2^			1.46	0.2658		
B^2^			13.33	0.0082 *		
C^2^			3.81	0.0917		
Lack of fit	0.3390	0.9180	0.0842	0.9651	1.35	0.4038

* Significant model terms.

**Table 5:** Composition of the selected optimized NIC–NLC formulations with different predicted and observed response values with prediction error.

Formula	Composition			Response	Predicted	Observed	Prediction Error (%)	Desirability	Drug Loading (%)	PDI ± SD
	A	B	C							
F1	30	3	4.17	R1 R2 R3	104.14 −14.16 93.7	111.18 −15.41 95.11	6.33 8.11 1.48	0.987	4.6%	0.251 ± 0.04
F2	30	3	4.36	R1 R2 R3	103.99 −14.20 93.56	106.04 −14.88 91.99	1.93 4.56 1.67	0.985	4.1%	0.273 ± 0.02
F3	30	3	4.79	R1	103.67	115.11	9.93	0.986	3.8%	0.279 ± 0.04
				R2	−14.17	−13.27	6.78			
				R3	93.21	94.61	1.47			

**Table 6:** Pharmacokinetic parameters of NIC in plasma and brain following IN and IV administration of the optimized NIC–NLC formula and NIC–SOL.

Organ/. tissue	AR	Formula	Pharmacokinetic Parameters					
			C max (µg/mL)	Tmax (h)	AUC (0-t) (µg.h/mL)	AUC (0-∞) (µg.h/mL)	Kel (h^−1^)	t_1/2_ (h)
**Plasma**	**IN**	NIC–NLC	1.68	2	8.65	9.43	0.218	3.17
		NIC–SOL	1.08	2	5.33	5.62	0.476	1.46
	IV	NIC–NLC	9.46	-	10.87	10.99	0.932	0.74
		NIC–SOL	9.84	-	7.71	7.76	0.903	0.77
**Brain**	IN	NIC–NLC	1.46	2	11.61	13.53	0.129	3.62
		NIC–SOL	0.75	2	2.78	2.96	0.461	1.51
	IV	NIC–NLC	1.37	1	8.04	8.42	0.291	2.38
		NIC–SOL	1.53	0.75	4.36	4.41	0.673	1.03

AR: Administration route, NIC–NLC (optimized formula), NIC–SOL (drug solution).

**Table 7:** One way ANOVA test for NIC pharmacokinetic plasma data following IN and IV administration from NLC formulation and solution in rats.

Parameter	Source	Sum of Squares	df	Mean Square	F	Significance
C max (µg/mL)	Between Groups	412.099	3	137.366	188923.61	<0.001
	Within Groups	0.015	20	0.001		
	Total	412.114	23			
AUC _(0–24)_ (µg·h/mL)	Between Groups	94.677	3	31.559	664514.72	
	Within Groups	0.001	20	0.000		
	Total	94.678	23			
AUC _(0–α)_ (µg·h/mL)	Between Groups	95.387	3	31.796	383621.32	
	Within Groups	0.002	20	0.000		
	Total	95.389	23			
Kel (h^−1^)	Between Groups	2.155	3	0.718	12655.19	
	Within Groups	0.001	20	0.000		
	Total	2.156	23			
t_1/2_ (h)	Between Groups	35.308	3	11.769	62118.63	
	Within Groups	0.004	20	0.000		
	Total	35.311	23			

**Table 8:** Post hoc statistical analysis of the calculated mean differences of different pharmacokinetic parameters in plasma.

Parameter	Group		Mean Difference	Confidence Interval	
				Lower	Upper
**C max (µg/mL)**	**NIC–NLC formula (IN)**	**NIC–SOL (IN)**	0.602667 *	0.55909	0.64624
		NIC–NLC (IV)	−7.780000 *	−7.82357	−7.73643
		NIC–SOL (IV)	−8.161667 *	−8.20524	−8.11809
AUC _(0−24)_ (µg.h/mL)		NIC–SOL (IN)	3.317333 *	3.30620	3.32847
		NIC–NLC (IV)	−2.220500 *	−2.23164	−2.20936
		NIC–SOL (IV)	0.937333 *	0.92620	0.94847
AUC _(0-α)_ (µg.h/mL)		NIC–SOL (IN)	3.810500 *	3.79579	3.82521
		NIC–NLC (IV)	−1.559667 *	−1.57438	−1.54495
		NIC–SOL (IV)	1.669833 *	1.65512	1.68455
Kel (h^−1^)		NIC–SOL (IN)	−0.2580333 *	−0.270207	−0.245860
		NIC–NLC (IV)	−0.7140333 *	−0.726207	−0.701860
		NIC–SOL (IV)	−0.6848667 *	−0.697040	−0.672693
t_1/2_ (h)		NIC–SOL (IN)	2.25133333 *	2.2290903	2.2735764
		NIC–NLC (IV)	2.97071667 *	2.9484736	2.9929597
		NIC–SOL (IV)	2.94116667 *	2.9189236	2.9634097

*: Significant.

**Table 9:** One way ANOVA test for NIC pharmacokinetic brain data following IN and IV administration from NLC formulation and solution in rats.

Parameter	Source	Sum of Squares	df	Mean Square	F	Significance
C max (µg/mL)	Between Groups	2.309	3	0.770	2710.53	<0.001
	Within Groups	0.006	20	0.000		
	Total	2.315	23			
Tmax (h)	Between Groups	7.781	3	2.594	-	
	Within Groups	0.000	20	0.000		
	Total	7.781	23			
AUC _(0−24)_ (µg·hr/mL)	Between Groups	280.430	3	93.477	62140.72	
	Within Groups	0.030	20	0.002		
	Total	280.460	23			
AUC _(0-α)_ (µg·hr/mL)	Between Groups	417.873	3	139.291	214847.56	
	Within Groups	0.013	20	0.001		
	Total	417.886	23			
K (h^−1^)	Between Groups	0.981	3	0.327	34365.89	
	Within Groups	0.000	20	0.000		
	Total	0.982	23			
t_1/2_ (h)	Between Groups	23.314	3	7.771	101699.49	
	Within Groups	0.002	20	0.000		
	Total	23.316	23			

**Table 10:** Post hoc statistical analysis of the calculated mean differences of different pharmacokinetic parameters in brain.

Parameter	Group		Mean Difference	Confidence Interval	
				Lower	Upper
C max (µg/mL)	**NIC–NLC formula (IN)**	NIC–SOL (IN)	0.711000 *	0.68377	0.73823
		NIC–NLC (IV)	0.091167 *	0.06393	0.11840
		NIC–SOL (IV)	−0.070333 *	−0.09757	−0.04310
AUC _(0–24)_ (µg.h/mL)		NIC–SOL (IN)	8.829833 *	8.76716	8.89251
		NIC–NLC (IV)	3.569333 *	3.50666	3.63201
		NIC–SOL (IV)	7.248333 *	7.18566	7.31101
AUC _(0–α)_ (µg.h/mL)		NIC–SOL (IN)	10.838333 *	10.79719	10.87948
		NIC–NLC (IV)	5.109333 *	5.06819	5.15048
		NIC–SOL (IV)	9.118833 *	9.07769	9.15998
Kel (h^−1^)		NIC–SOL (IN)	−0.3319667 *	−0.336953	−0.326981
		NIC–NLC (IV)	−0.1599000 *	−0.164886	−0.154914
		NIC–SOL (IV)	−0.5442167 *	−0.549203	−0.539231
t_1/2_ (h)		NIC–SOL (IN)	2.11368333 *	2.0995572	2.1278095
		NIC–NLC (IV)	1.24011667 *	1.2259905	1.2542428
		NIC–SOL (IV)	2.59192500 *	2.5777989	2.6060511

*: Significant.

**Table 11:** Average LC, ZP, PS, and PDI of the optimized NIC–NLC formula after storage for one months at different storage condition in comparison to fresh formula.

Storage Conditions	LC ± SD (%)	ZP ± SD (mV)	PS ± SD (nm)	PDI ± SD
**Fresh**	4.6 ± 0.34	−15.41 ± 0.71	111.18 ± 4.71	0.251 ± 0.04
**25 °C**	4.3 ± 0.31	−19.45 ± 0.92	132.65 ± 5.29	0.273 ± 0.01
**4 °C**	4.5 ± 0.21	−15.89 ± 0.38	117.42 ± 2.11	0.257 ± 0.02

LC: Loading capacity, ZP: Zeta potential, PS: Particle size, PDI: Polydispersity index.

## Data Availability

Not applicable.
